# Supplementary material for: Loss of polarity by Cdc42 depletion and oncogenic Kras activation in the mouse intestinal epithelia leads to a necrotizing enterocolitis (NEC)-like disease
Source: Nat Commun. 2026 Mar 18;17:4852. doi: 10.1038/s41467-026-70677-9 (PMC13223224; doi:10.1038/s41467-026-70677-9)
Supplement: Supplementary file 1 — Supplementary Information [file 41467_2026_70677_MOESM1_ESM.pdf]

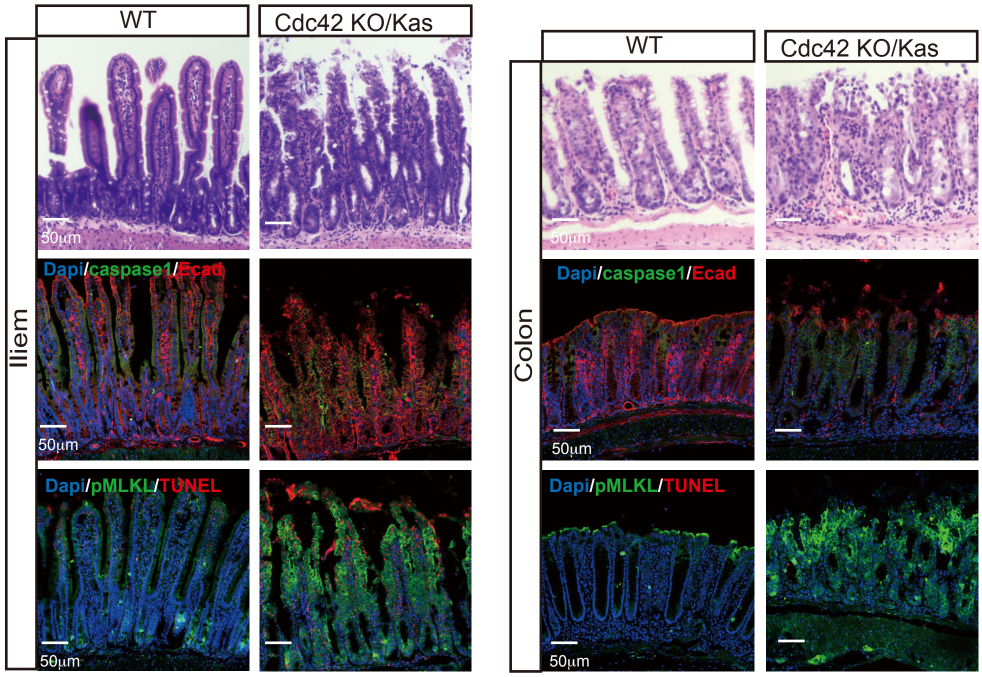

Supplemental Figure 1

Supplemental Figure 1. Cdc42 KO combined with oncogenic Kras G12D mutation causes necroptosis and disrupted epithelium in the ileum and colon. Related to Figure 1.

Three to four-month-old mice were injected with TAM once per day for 3 days, and then were sacrificed at 96 h after the 1st TAM injection. Representative images of H&E and immunofluorescence staining of ileum (left) and colon (right) sections are shown. Data are representative of at least three independent experiments. Scale bars, 50 μm.

A

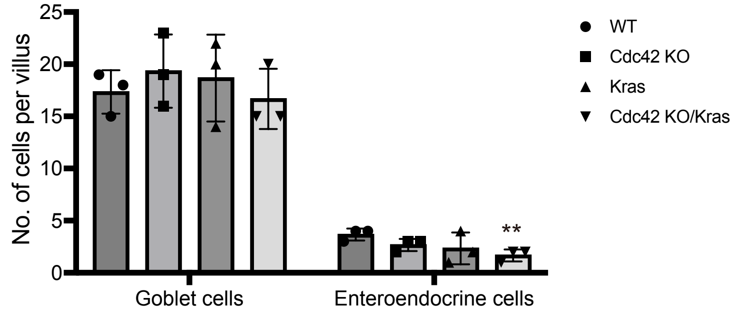

B

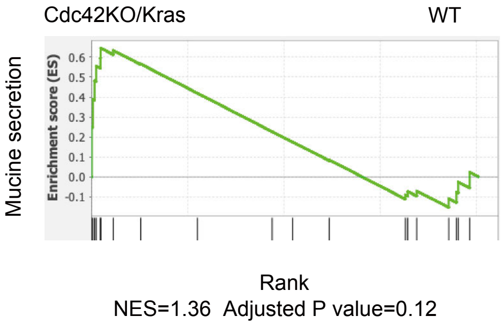

NES=1.36 Adjusted P value=0.12

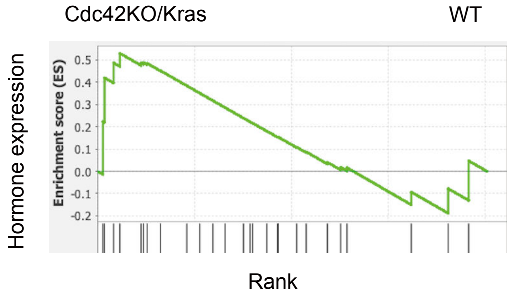

NES=1.12 Adjusted P value=0.30

	SYMBOL	RANK IN GENE LIST	RANK METRIC SCORE	RUNNING ES	CORE ENRICHMENT
1	Muc3	25	3.171	0.2510	Yes
2	Zg16	85	1.777	0.3893	Yes
3	Tff3	177	1.212	0.4812	Yes
4	Lypd8	255	0.987	0.5558	Yes
5	Muc13	477	0.646	0.5962	Yes
6	Fcgbp	494	0.627	0.6453	Yes
7	Muc4	1148	0.262	0.6337	No
8	Muc5b	2570	0.039	0.5660	No
9	St6galnac1	5520	0.000	0.4192	No
10	Muc5ac	9396	−0.006	0.2267	No
11	Muc1	10459	−0.015	0.1750	No
12	Clca1	12358	−0.055	0.0849	No
13	Ergic2	16314	−0.272	−0.0904	No
14	Ern2	16439	−0.283	−0.0741	No
15	Ergic3	16905	−0.326	−0.0714	No
16	Ryk	18570	−0.562	−0.1096	No
17	Cftr	18967	−0.661	−0.0767	No
18	Agr2	19064	−0.692	−0.0265	No
19	Ergic1	19652	−0.982	0.0224	No

	SYMBOL	RANK IN GENE LIST	RANK METRIC SCORE	RUNNING ES	CORE ENRICHMENT
1	Sct	287	1.041	0.2234	Yes
2	Slc15a1	371	0.876	0.4193	Yes
3	Pcsk1	841	0.395	0.4862	Yes
4	Cpe	1163	0.259	0.5293	Yes
5	Gcg	2253	0.046	0.4856	No
6	Fabp5	2378	0.036	0.4877	No
7	Ffar4	2567	0.025	0.4841	No
8	Pyy	3256	0.006	0.4511	No
9	Nts	4614	0.001	0.3836	No
10	Ffar1	5277	0.000	0.3507	No
11	Gpr119	5960	0.000	0.3167	No
12	Tas1r2	6583	0.000	0.2857	No
13	Sst	7535	−0.001	0.2386	No
14	Gast	7876	−0.002	0.2221	No
15	Tas1r1	8008	−0.002	0.2159	No
16	Lmx1a	8736	−0.004	0.1805	No
17	Pcsk1n	9298	−0.005	0.1536	No
18	Chga	9330	−0.005	0.1532	No
19	Tas1r3	10276	−0.009	0.1083	No
20	Ghrl	10768	−0.014	0.0870	No
21	Tph1	11858	−0.032	0.0401	No
22	Gip	12546	−0.051	0.0176	No
23	Cd36	12870	−0.063	0.0159	No
24	Cck	16198	−0.248	−0.0931	No
25	Chgb	18101	−0.482	−0.0779	No
26	Slc5a1	19157	−0.778	0.0470	No

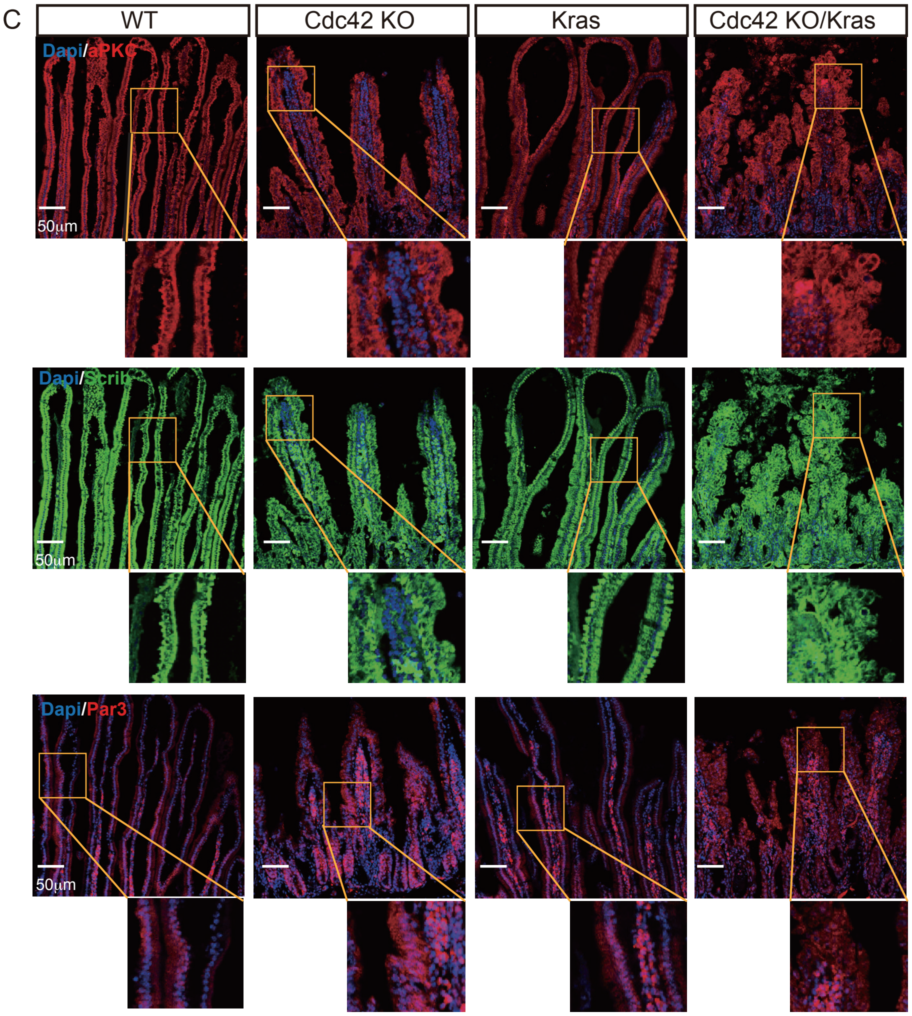

Supplemental Figure 2

Supplemental Figure 2. Cdc42 KO combined with oncogenic Kras G12D does not significantly affect goblet cell or enteroendocrine cell population and function in the small intestine, while polarity is disrupted. Related to Figure 2.

(A) Quantification of number of goblet cells/enteroendocrine per villus. Data are mean ± SD; two-tailed unpaired Student's t-test, **p= 0.013, n = 3 mice for each group tested. Source data are provided as a Source Data file.

(B) Top: GSEA pathway enrichment maps for mucine secretion genes in goblet cells, and hormone secretion genes in enteroendocrine cells, Cdc42 KO/Kras vs WT. Bottom: The complete list of genes for GSEA pathway enrichment.

(C) Representative images of immunofluorescence staining of duodenal sections are shown. Data are representative of at least three independent experiments. Scale bars, 50 μm.

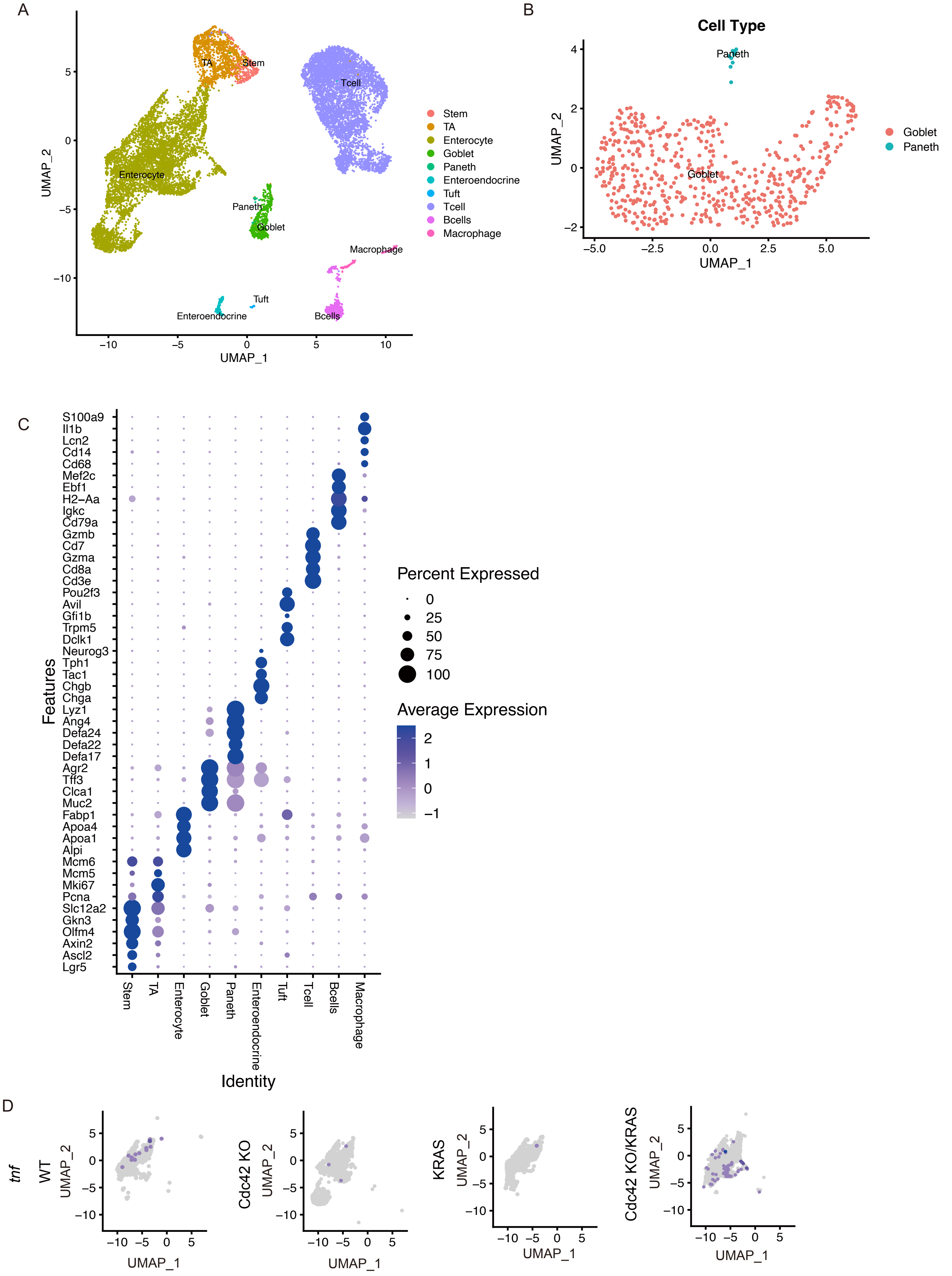

A
B
Cell Type
C
D
UMAP_1
UMAP_2
Stem
TA
Enterocyte
Goblet
Paneth
Enteroendocrine
Tuft
Tcell
Bcells
Macrophage
Features
Identity
Percent Expressed
0
25
50
75
100
Average Expression
S100a9
Il1b
Lcn2
Cd14
Cd68
Mef2c
Ebf1
H2−Aa
Igkc
Cd79a
Gzmb
Cd7
Gzma
Cd8a
Cd3e
Pou2f3
Avil
Gfi1b
Trpm5
Dclk1
Neurog3
Tph1
Tac1
Chgb
Chga
Lyz1
Ang4
Defa24
Defa22
Defa17
Agr2
Tff3
Clca1
Muc2
Fabp1
Apoa4
Apoa1
Alpi
Mcm6
Mcm5
Mki67
Pcna
Slc12a2
Gkn3
Olfm4
Axin2
Ascl2
Lgr5
tnf
WT
Cdc42 KO
KRAS
Cdc42 KO/KRAS

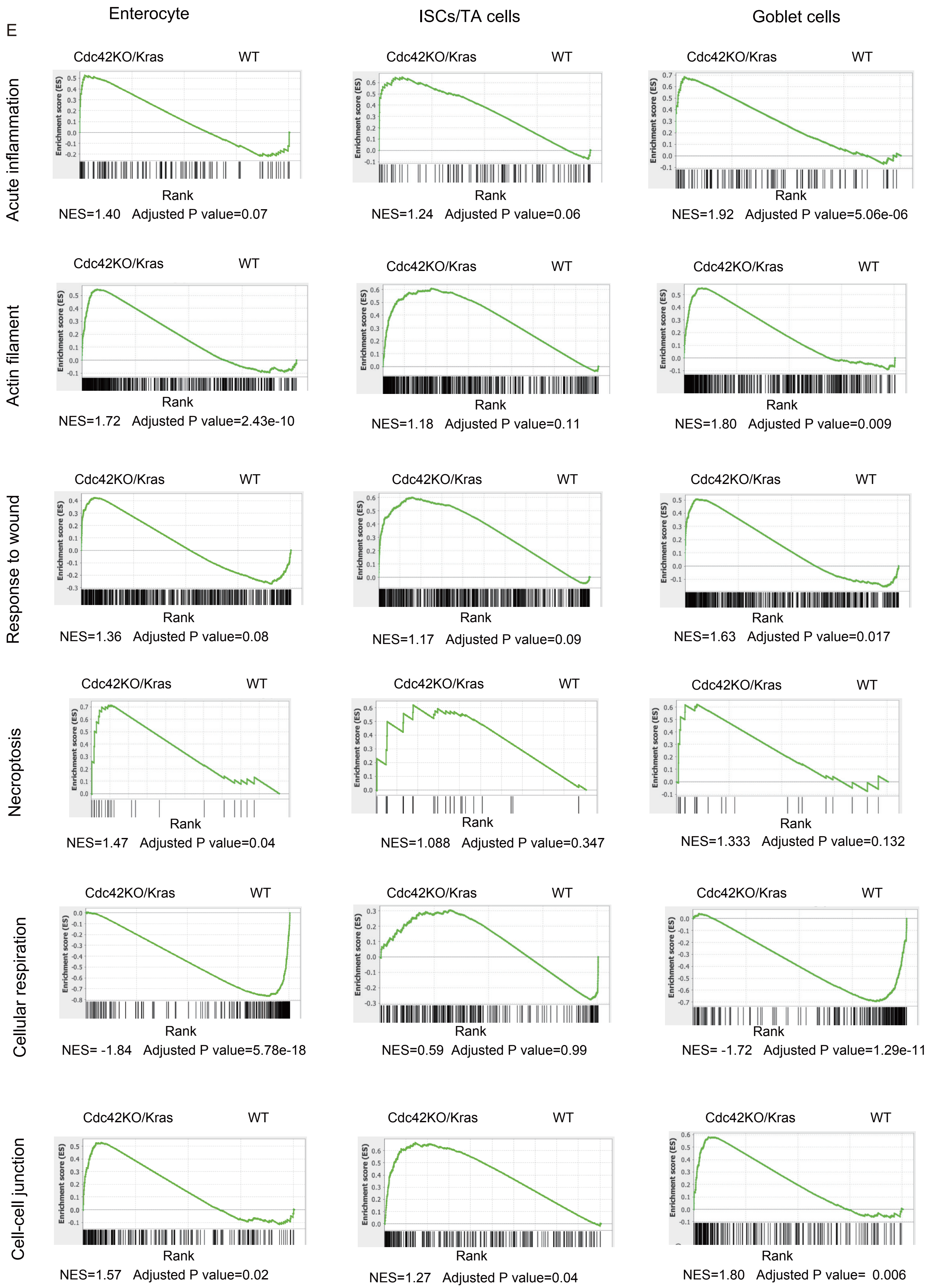

Supplemental Figure 3

Supplemental Figure 3. UMAP with annotations and GSEA analyses of single-cell RNA-seq in three major cell subtypes of intestinal populations. Related to Figure 3.

(A) Single-cell RNA-seq analyses of isolated single cells from duodenal villis/crypts by UMAP clustering.

(B) The highly similar goblet and Paneth cells were further differentiated by subsetting and reclustering the cell population containing both cell types in an unbiased manner as above. This resulted in a small group of cells separated from the rest that were enriched with canonical Paneth markers, Lyz1, Defa17, Defa22, Defa24, and Ang4. These cells were annotated as Paneth cells and the rest as goblet cells

(C) Dot plot showing the major marker genes for each cell type shown in the UMAP.

(D) Expression level of necrosis marker *tnf* in all intestinal cell type clusters.

(E) GSEA pathway enrichment maps for acute inflammation, necroptosis, actin filament, cellular respiration, response to wound and cell-cell junction pathways in three different subtypes of intestinal cells: enterocytes, ISCs/TA cells, goblet cells; Cdc42 KO/Kras vs WT.

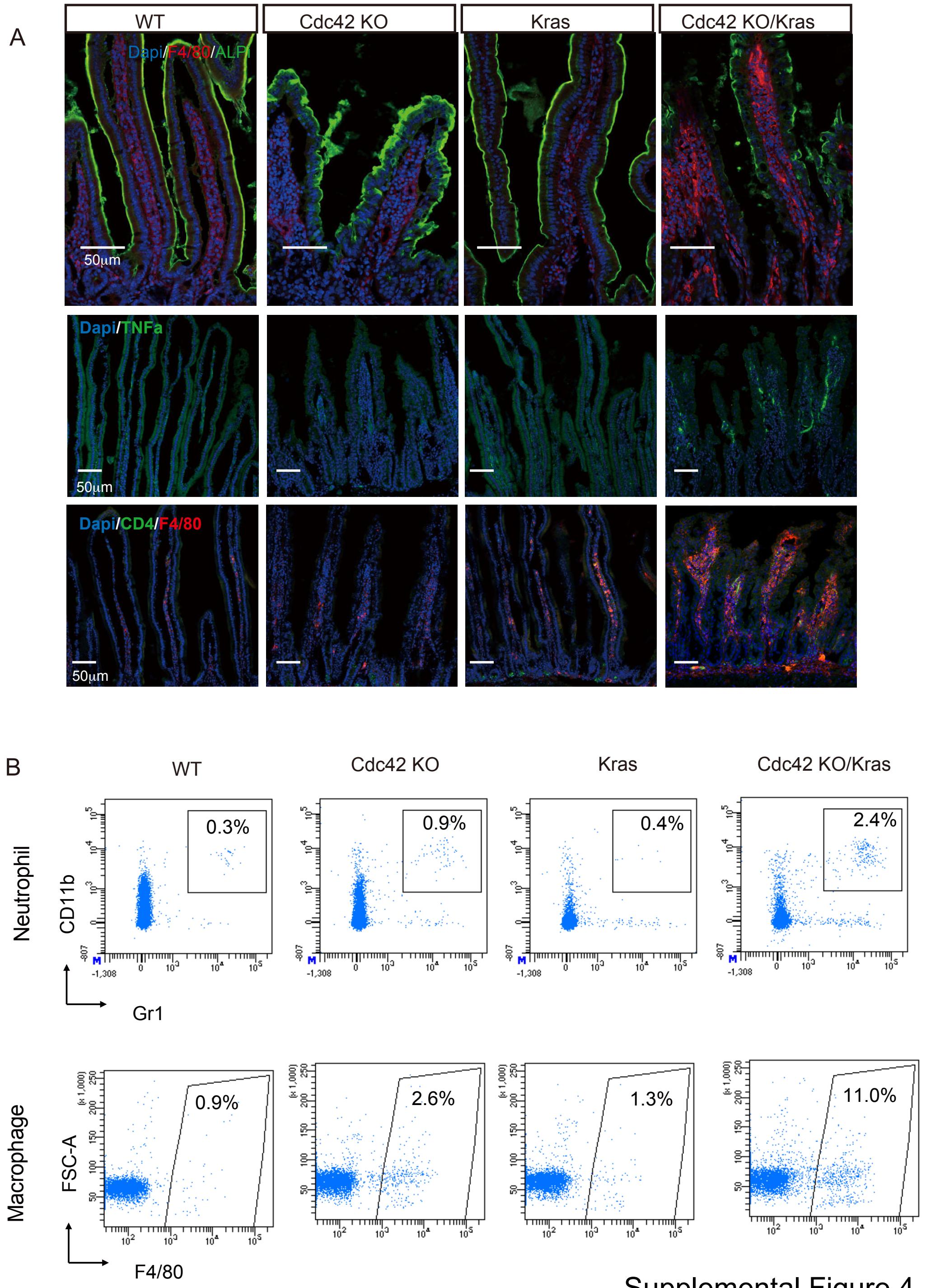

Supplemental Figure 4

Supplemental Figure 4. Cdc42 KO/Kras G12D mice have increased macrophages and neutrophils in small intestine. Related to Figure 4.

(A) Representative images of immunofluorescence staining of duodenal sections. Data are representative of at least three independent experiments. Scale bars, 50 μm.

(B) Representative flow cytometry plots showing the percentages of neutrophil and macrophage among live CD45+ cells in single-cell suspensions from duodenal villus/crypts. Data are mean ± SD. The number within the gate represents the average percentage of the indicated population.

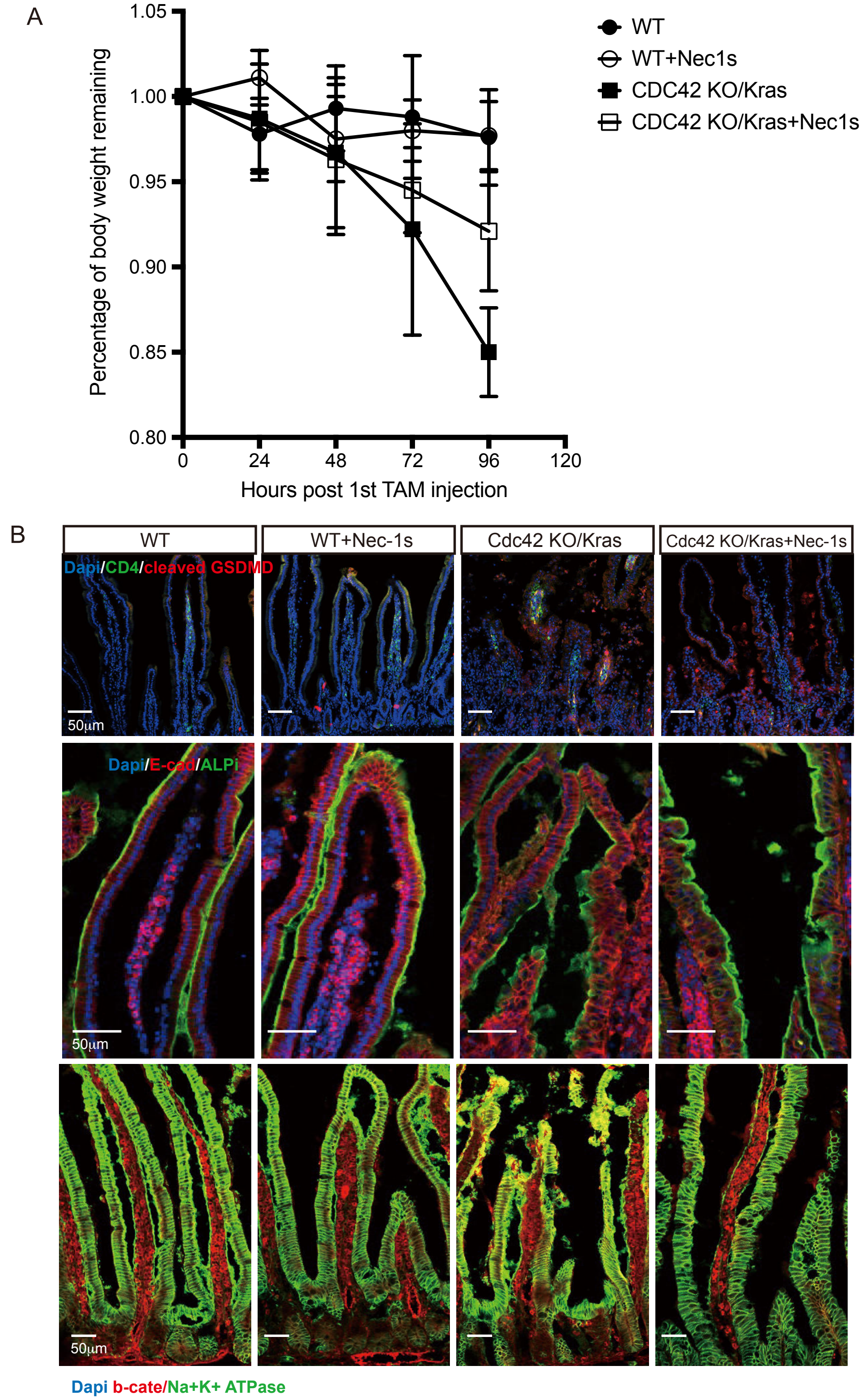
A
Percentage of body weight remaining
1.05
1.00
0.95
0.90
0.85
0.80
0
24
48
72
96
120
Hours post 1st TAM injection
WT
WT+Nec1s
CDC42 KO/Kras
CDC42 KO/Kras+Nec1s
B
WT
WT+Nec-1s
Cdc42 KO/Kras
Cdc42 KO/Kras+Nec-1s
Dapi/CD4/cleaved GSDMD
50µm
Dapi/E-cad/ALPi
50µm
50µm
Dapi b-cate/Na+K+ ATPase

C

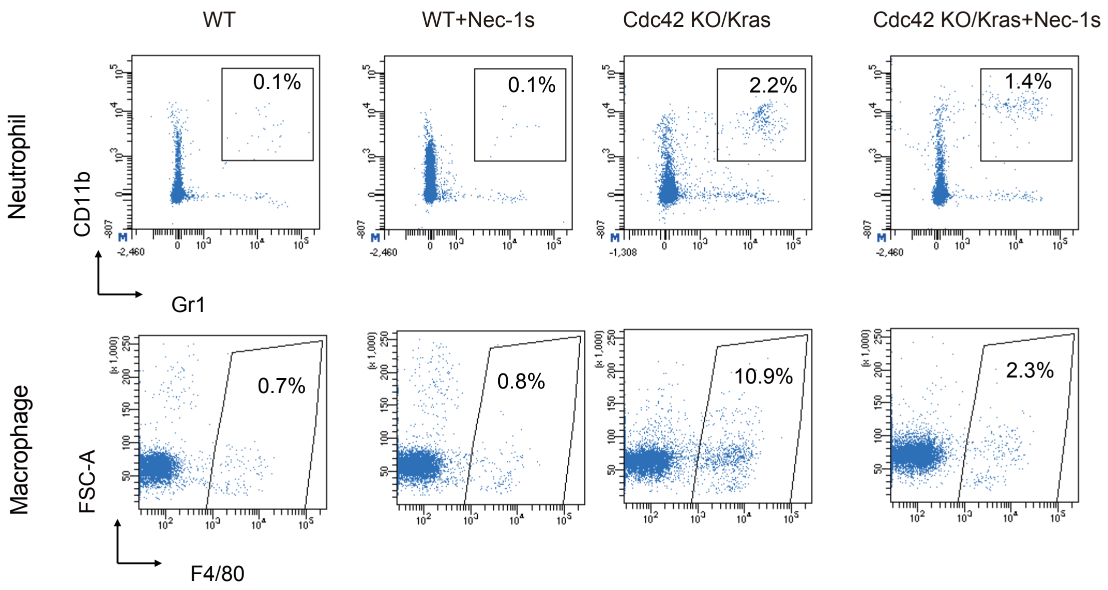

D

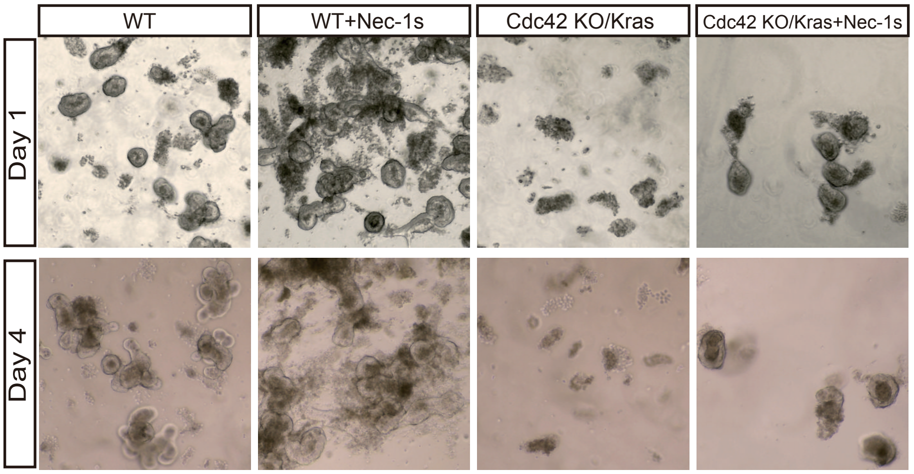

Supplemental Figure 5

Supplemental Figure 5. Necroptosis inhibitor Nec-1s rescues body weight, restores enterocyte population, and reduces inflammatory cells, but only partially rescues ISC clonal function in Cdc42 KO/Kras G12D mice. Related to Figure 5.

(A) Percentage of body weight remaining at 24-120 h after 1st TAM injection. Data are mean ± SD, WT n=4 mice, WT+Nec1s n=3 mice, Cdc42 KO/Kras n=3 mice, Cdc42 KO/Kras+Nec1s n=6 mice. Source data are provided as a Source Data file.

(B) Representative images of immunofluorescence staining of duodenal sections. Data are representative of at least three independent experiments. Scale bars, 50 μm.

(C) Representative flow cytometry plots showing the percentages of neutrophil and macrophage among live CD45+ cells in single-cell suspensions from duodenal villus/crypts. Data are mean ± SD. Data are representative of at least three independent experiments. The number within the gate represents the average percentage of the indicated population.

(D) Representative images of growth of WT, WT+Nec-1s, Cdc42 KO/Kras and Cdc42 KO/Kras + Nec-1s enteroids after 1 day and 4 days of culture.

A

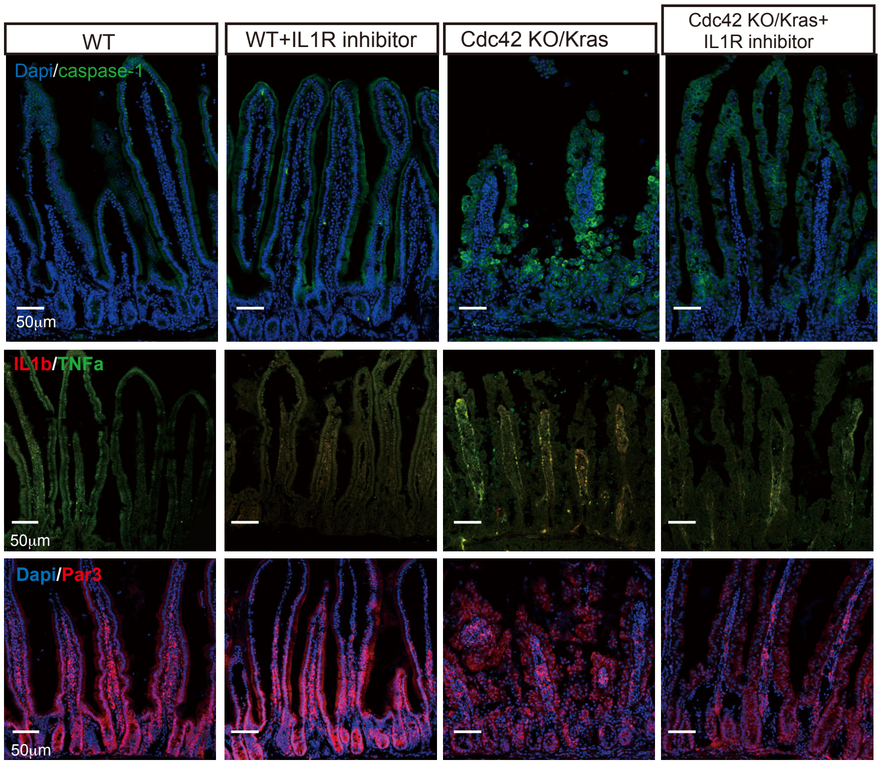
WT
WT+IL1R inhibitor
Cdc42 KO/Kras
Cdc42 KO/Kras+ IL1R inhibitor
Dapi/caspase-1
50μm
IL1b/TNFa
50μm
Dapi/Par3
50μm

B

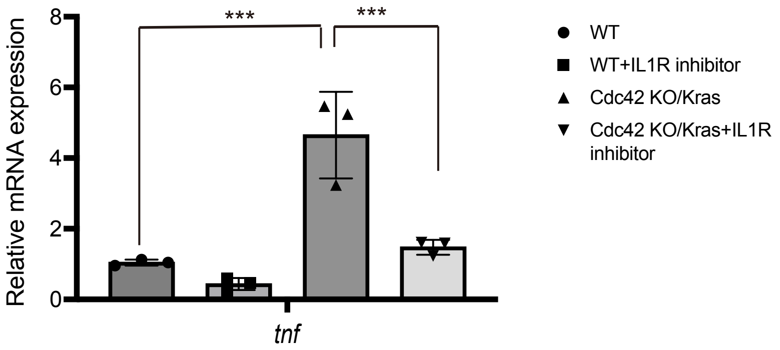
Relative mRNA expression
8
6
4
2
0
***
***
tnf
WT
WT+IL1R inhibitor
Cdc42 KO/Kras
Cdc42 KO/Kras+IL1R inhibitor

C

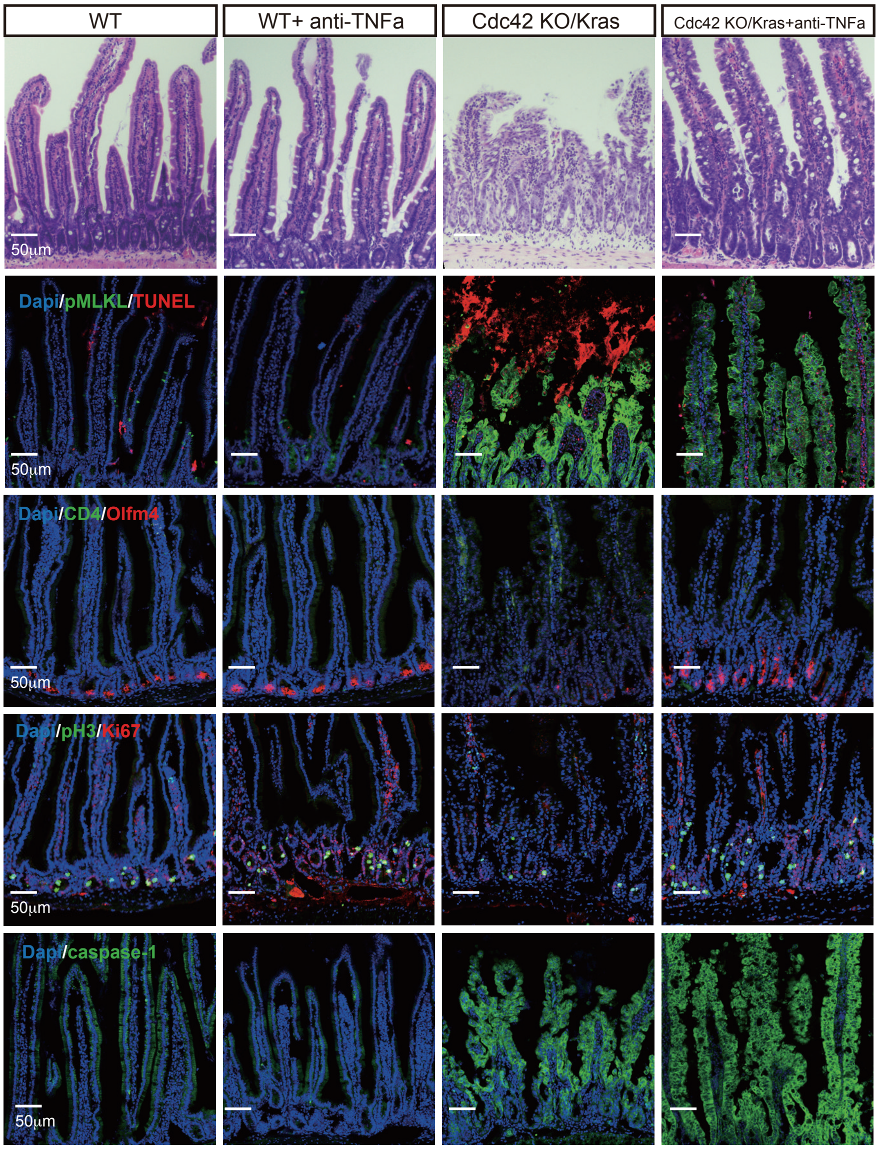

Supplemental Figure 6

Supplemental Figure 6. IL-1R inhibition reduces caspase-1 expression and the necrosis marker Tnf in Cdc42 KO/Kras G12D mice. Anti-TNFa rescues Cdc42 KO/Kras G12D intestinal defects. Related to Figure 6.

(A) Representative images of immunofluorescence staining of duodenal sections. Data are representative of at least three independent experiments. Scale bars, 50 μm.

(B) Quantification of relative Tnf mRNA expression. Data are mean ± SD; two-tailed unpaired Student's t-test, ***p= 0.0002, ***p= 0.0004, n = 3 mice for each group tested. Source data are provided as a Source Data file.

(C) Representative images of H&E and immunofluorescence staining of duodenal sections. Data are representative of two independent experiments. Scale bars, 50 μm.

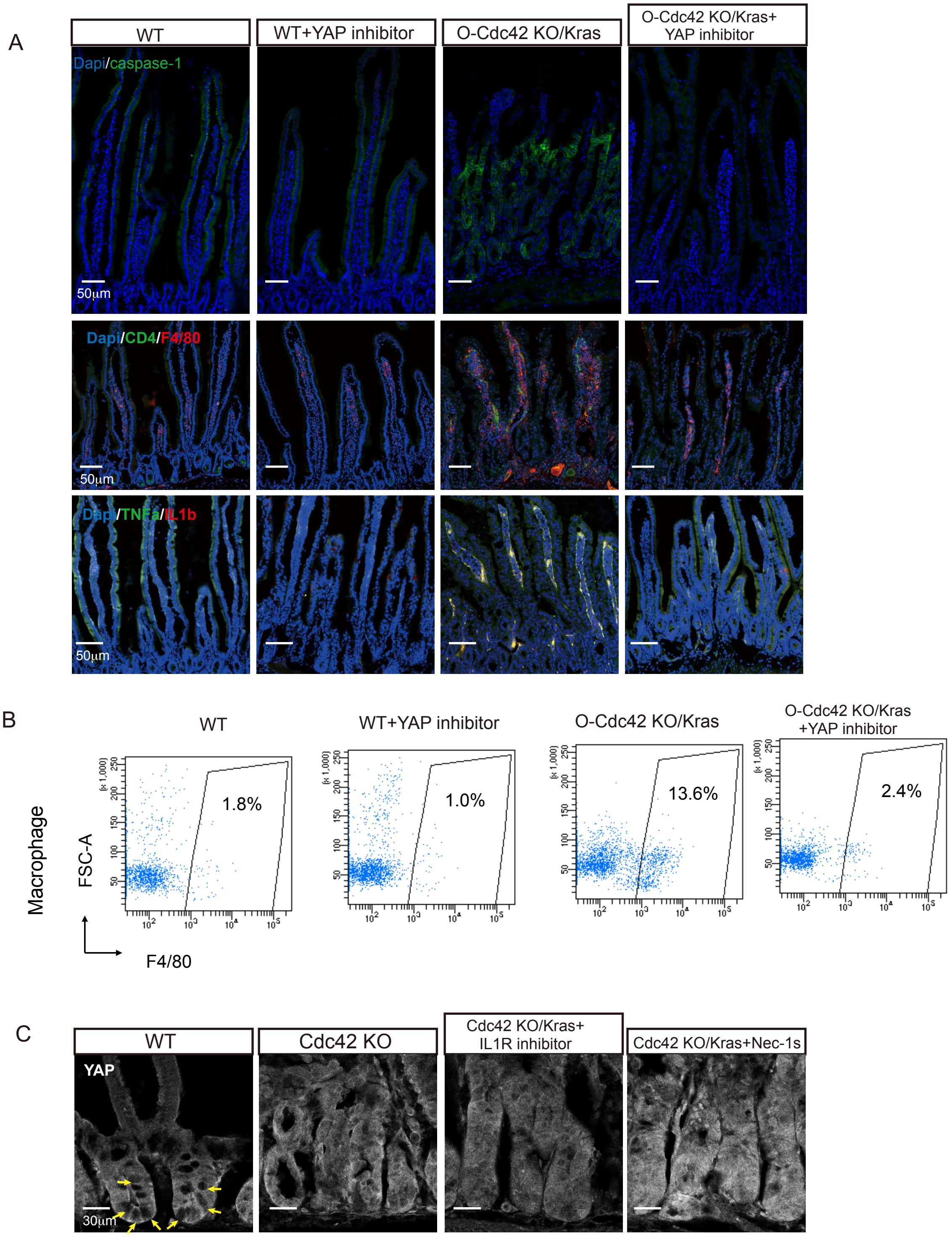

Supplemental Figure 7

Supplemental Figure 7. YAP inhibition reduces caspase-1 expression and inflammatory response in ISC-target Cdc42 KO/Kras G12D mice, while IL1R or necroptosis inhibition has no effect on nuclear YAP expression. Related to Figure 7.

(A) Representative images of immunofluorescence staining of duodenal sections. Data are representative of at least three independent experiments. Scale bars, 50 μm.

(B) Representative flow cytometry plots showing the percentage of macrophage among live CD45+ cells in single-cell suspensions from duodenal villus/crypts.

(C) Representative images of YAP immunofluorescence staining of duodenal sections. Data are representative of two independent experiments. Arrow pointing to the nuclear YAP staining; Scale bars, 30 μm.

A

A	B	Neither	A Not B	B Not A	Both	g2 Odds Rat	p-Value	q-Value	Tendency
KRAS	SCRIB	703	384	55	9	-1.739	<0.001	0.001	Mutual exclusivity
KRAS	DLG5	714	382	44	11	-1.098	0.014	0.043	Mutual exclusivity
KRAS	CDC42	751	390	7	3	-0.277	0.537	0,564	Mutual exclusivity

B

Patient Overlay

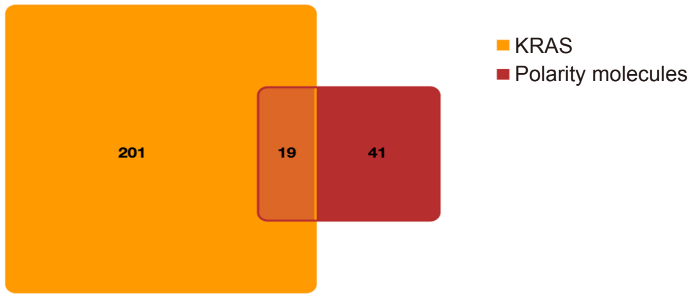

Supplemental Figure 8

Supplemental Figure 8. Mutations of KRAS and polarity molecules are mutually exclusive in colon cancer patients.

(A) The relationship between KRAS mutations and other polarity molecules mutations in TCGA colon cancer patients datasets;

(B) Patient overlay between KRAS mutation and other polarity molecules mutations;

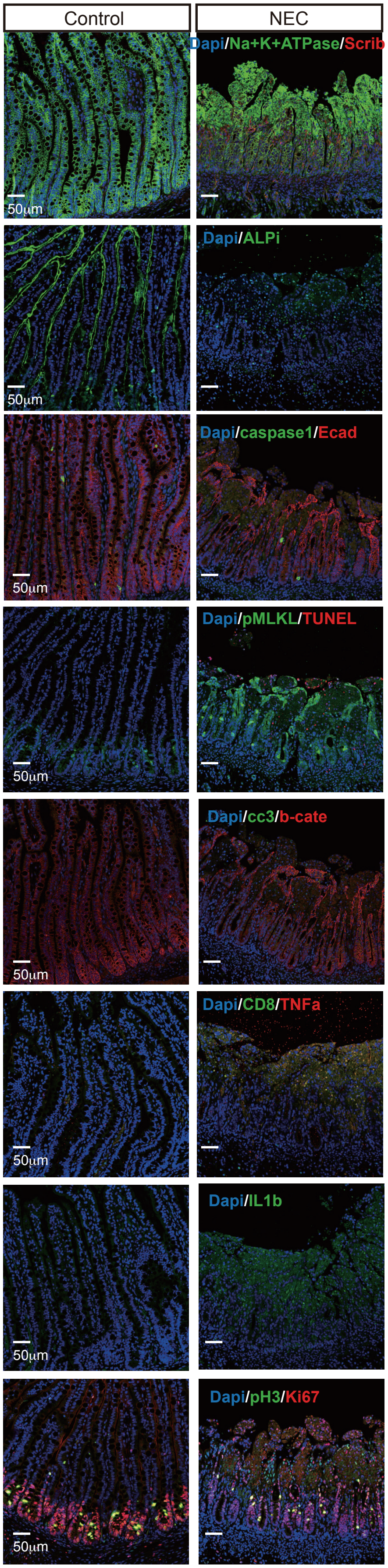

Supplemental Figure 9

Supplemental Figure 9. Immunophenotypes of human NEC patient samples resemble that of mouse Cdc42 KO/Kras G12D intestinal defects.

Representative images of immunofluorescence staining of duodenal sections. Data are representative of one human NEC patient and one healthy human patient. Scale bars, 50 μm.
