# Supplementary material for: Loss of polarity by Cdc42 depletion and oncogenic Kras activation in the mouse intestinal epithelia leads to a necrotizing enterocolitis (NEC)-like disease
Source: Nat Commun. 2026 Mar 18;17:4852. doi: 10.1038/s41467-026-70677-9 (PMC13223224; doi:10.1038/s41467-026-70677-9)
Supplement: Supplementary file 2 — Description of Additional Supplementary Files [file 41467_2026_70677_MOESM2_ESM.pdf]

Description of Additional Supplementary Files

Supplementary Data 1- Reagents or resources used in the manuscript
