# Supplementary material for: Loss of polarity by Cdc42 depletion and oncogenic Kras activation in the mouse intestinal epithelia leads to a necrotizing enterocolitis (NEC)-like disease
Source: Nat Commun. 2026 Mar 18;17:4852. doi: 10.1038/s41467-026-70677-9 (PMC13223224; doi:10.1038/s41467-026-70677-9)
Supplement: Supplementary file 3 — Supplementary Data 1 [file 41467_2026_70677_MOESM3_ESM.docx]

**Reagents or resources used in the manuscript**

| REAGENT or RESOURCE | SOURCE | IDENTIFIER |
| --- | --- | --- |
| Antibodies | | |
| Rabbit anti- YAP (D8H1X) | Cell Signaling | Cat# 14074S,  Lot# 4,  Clone D8H1X |
| Rabbit anti- cleaved caspase 3 | Cell Signaling | Cat# 9664,  Lot# 22,  Clone 5A1E |
| Rabbit anti- phospho-histone H3 | Cell Signaling | Cat# 3377,  Lot# 9,  Clone D2C8 |
| Rabbit anti- Olfm4 | Cell Signaling | Cat# 39141S,  Lot# 1,  Clone D6Y5A |
| Mouse anti- E-cadherin | BD Biosciences | Cat# 610182,  Lot# 3138351 |
| Mouse anti- beta-catenin | BD Biosciences | Cat# 610153,  Lot# 2038930 |
| Mouse anti- alpha E-catenin | Santa Cruz | Cat# SC-9988,  Lot# B0724,  Clone G-11 |
| Rabbit anti- ZO-1 | Invitrogen | Cat# 61-7300,  Lot# YC368286 |
| Rabbit anti- Na+K+ATPase | Abcam | Cat# ab76020,  Lot# GR3375102-6,  Clone EP1845Y |
| Rat anti- Ki67 | Invitrogen | Cat# 14-5698-80,  Lot# 2755290,  Clone SolA15 |
| Rabbit anti- Lysozyme | Dako | Cat# A0099,  Lot# 41311054 |
| Rabbit anti- Cdc42 | Cell Signaling | Cat# 2466S,  Lot# 6,  Clone 11A11 |
| Rabbit anti- beta-actin | Cell Signaling | Cat# 4970S,  Lot# 18,  Clone 13E5 |
| Rabbit anti- p44/42 MAPK (Erk1/2) | Cell Signaling | Cat# 4695S,  Lot# 28,  Clone 137F5 |
| Rabbit anti- Phospho-p44/42 MAPK (Erk1/2) | Cell Signaling | Cat# 4370S,  Lot# 28,  Clone D13.14.4E |
| Rabbit anti- AKT | Cell Signaling | Cat# 9272S,  Lot# 28 |
| Rabbit anti- Phospho-AKT (Thr308) | Cell Signaling | Cat# 4056S,  Lot# 23,  Clone 244F9 |
| Rabbit anti- ALPi | Invitrogen | Cat# PA5-22210,  Lot# ZD4287935 |
| Rabbit anti- Mucin 2 | Abcam | Cat# ab272692,  Lot# GR3374627-20,  Clone EPR23479-47 |
| Rabbit anti- Chromogranin A | Abcam | Cat# ab45179,  Lot# GR3417483-2 |
| Rabbit anti- caspase-1 (E2Z1C) | Cell Signaling | Cat# 24232S,  Lot# 4,  Clone E2Z1C |
| Rabbit anti- RIP (D94C12) | Cell Signaling | Cat# 3493S,  Lot# 6,  Clone D94C12 |
| Rabbit anti- pMLKL (phosphor S345) | Abcam | Cat# ab196436,  Lot# 1001237-28,  Clone EPR9515(2) |
| Rat anti- F4/80 (for IF) | Invitrogen | Cat# 14-4801-82,  Lot# 2488480,  Clone BM8 |
| Goat anti- TNF-alpha | R&D systems | Cat# AF-410-NA,  Lot# NQ2824121 |
| Rabbit anti- IL-1-beta (D3U3E) | Cell Signaling | Cat# 12703T,  Lot# 6,  Clone D3U3E |
| Rabbit anti- Par3 | Proteintech | Cat# 11085-1-AP,  Lot# 00097544 |
| Sheep anti-PCK (iota / lambda / zeta) | R&D systems | Cat# AF4465,  Lot# ZUR012507A |
| Rat anti- CD4 (RM4-5) | Cell Signaling | Cat# 96127S,  Lot# 8,  Clone RM4-5 |
| Rabbit anti- Cleaved Gasdermin D (Asp275) | Cell Signaling | Cat# 36425T,  Lot# 4,  Clone E7H9G |
| Mouse anti- Scrib | Santa Cruz | Cat# sc-374139,  Lot# A1223,  Clone D-2 |
| Anti-mouse IgG, HRP-linked Antibody | Cell Signaling | Cat# 7076S,  Lot# 32 |
| Anti-rabbit IgG, HRP-linked Antibody | Cell Signaling | Cat# 7074S,  Lot# 32 |
| Alexa Fluor 488, Goat anti Rabbit IgG | Invitrogen | Cat# A11008,  Lot# 2897813 |
| Alexa Fluor 568, Goat anti Rabbit IgG | Invitrogen | Cat# A11011,  Lot# 2192277 |
| Alexa Fluor 633, Goat anti Rabbit IgG | Invitrogen | Cat# A21070,  Lot# 2432056 |
| Alexa Fluor 488, Goat anti Mouse IgG | Invitrogen | Cat# A21121,  Lot# 2180679 |
| Alexa Fluor 568, Goat anti Mouse IgG | Invitrogen | Cat# A11004,  Lot# 2855933 |
| Alexa Fluor 633, Goat anti Mouse IgG | Invitrogen | Cat# A21052,  Lot# 1964362 |
| FITC Rat Anti-Mouse CD45 (for flow) | BD Biosciences | Cat# 553080,  Lot# 2301642,  Clone 30-F11 |
| APC-Cy7 Rat Anti-CD11b (for flow) | BD Biosciences | Cat# 557657,  Lot# 2329160,  Clone M1/70 |
| PE Rat Anti-Mouse F4/80 (for flow) | BD Biosciences | Cat# 565410,  Lot# 2165051,  Clone T45-2342 |
| BV510 Rat Anti-Mouse Ly-6G and Ly-6C (for flow) | BD Biosciences | Cat# 563040,  Lot# 2202863,  Clone RB6-8C5 |
|  |  |  |
| Primers for qPCR | | |
| *cdc42* | Thermo Fisher | Mm01194005_g1 |
| *beta-actin* | Thermo Fisher | Mm02619580_g1 |
| *lgr5* | Thermo Fisher | Mm00438890_m1 |
| *cyclin D1 (ccnd)* | Thermo Fisher | Mm00432359_m1 |
| *IL-1β* | Thermo Fisher | Mm00434228_m1 |
| *tnf* | Thermo Fisher | Mm00443258_m1 |
| *defa5* | Thermo Fisher | Mm00651548_g1 |
|  |  |  |
| Chemicals, Peptides, and Recombinant Proteins | | |
| Tamoxifen | Cayman Chemical | 13258 |
| anakinra | Sobi | KINERET |
| Verteporfin | AdooQ BioScience | A12658 |
| Anti-TNF-alpha antibody | Bio X Cell | BE0058 |
| Pierce™ Phosphatase Inhibitor Mini Tablets | Thermo Fisher | A32957 |
| Simple Stop™ 1 Phosphatase Inhibitor Cocktail | Gold Biotechnology | GB450 |
| complete™, Mini, EDTA-free Protease Inhibitor Cocktail | Roche | 11836170001 |
| Alcian Blue | Sigma | A3157 |
| DAPI (4',6-Diamidino-2-Phenylindole, Dihydrochloride) | Invitrogen | D1306 |
|  |  |  |
| Critical Commercial Assays | | |
| RNeasy Micro Kit | Qiagen | 74004 |
| High-Capacity cDNA Reverse Transcription Kit | Applied Biosystems | 4368814 |
| TaqMan™ Gene Expression Master Mix | Applied Biosystems | 4369514 |
|  |  |  |
| Deposited Data | | |
| Raw and analyzed data | GEO | GSE294390 (GSM8903735, GSM8903736, GSM8903737, GSM8903738) |
|  |  |  |
| Software and Algorithms | | |
| ImageJ | ^22^ | https://imagej.nih.gov/ij/ |
| Prism | GraphPad Software | https://www.graphpad.com/scientific-software/prism/ |
| Cell Ranger 1.3.0 | 10x Genomics | https://support.10xgenomics.com/single-cell-gene-expression/software/downloads/1.3 |
| R |  | https://www.r-project.org/ |
|  |  |  |
| Experimental Models: Organisms/Strains | | |
| Mouse: Olfm4-IRES-eGFPCreERT2 | Reference^15^ | N/A |
| Mouse: Cdc42^flox/flox^ | Reference^4^ | N/A |
| Mouse: villin-creERT2 | Reference^4^ | N/A |
|  |  |  |
| Mouse Strains | Short name |  |
| villin-creERT2; Cdc42^+/flox^ | WT |  |
| villin-creERT2; Cdc42^flox/flox^ | Cdc42 KO |  |
| villin-creERT2; Kras^LSL-G12D +^ | Kras |  |
| villin-CreER; Cdc42^flox/flox^; Kras^LSL-G12D +^ | Cdc42 KO/Kras |  |
| Olfm4-IRES-eGFPCreERT2; Cdc42^+/flox^ | O-WT |  |
| Olfm4-IRES-eGFPCreERT2; Cdc42^flox/flox^ | O-Cdc42 KO |  |
| Olfm4-IRES-eGFPCreERT2; Kras^LSL-G12D +^ | O-Kras |  |
| Olfm4-IRES-eGFPCreERT2; Cdc42^flox/flox^; Kras^LSL-G12D +^ | O- Cdc42 KO/Kras |  |
|  |  |  |
| Other | | |
| TUNEL (In Situ Cell Death Detection Kit, TMR red) | Roche | Cat#12156792910 |
